# Ambient data analytic on indoor environment monitoring for office buildings in hot and humid climates

**DOI:** 10.1016/j.dib.2019.104534

**Published:** 2019-09-17

**Authors:** Afiqah Ngah Nasaruddin, Boon Tuan Tee, Mohd Tahir Musthafah, Md Eirfan Safwan Md Jasman

**Affiliations:** a Fakulti Kejuruteraan Mekanikal, Universiti Teknikal Malaysia Melaka, Hang Tuah Jaya, 76100 Durian Tunggal, Melaka, Malaysia; b Centre for Advanced Research on Energy, Universiti Teknikal Malaysia Melaka, Hang Tuah Jaya, 76100 Durian Tunggal, Melaka, Malaysia

**Keywords:** Monitoring system, Indoor environment, Thermal comfort

## Abstract

The rapid development of open source developmental boards incorporating microcontrollers on printed circuit boards has offered many alternatives in creating feasible, low cost indoor environment monitoring and controlling platforms. Data are collected and stored in predetermined locations throughout a series of communication activities between a network of active sensors and their processing units. However, the issue of data precision and accuracy are of real concern for generating baseline information. Therefore, with that in mind, the purpose of this paper is to accentuate an insightful trend of retrieving indoor environment data (temperature and relative humidity) for an office building in a hot and humid climate condition. The indoor parameters were monitored using a combination of a single board microcontroller with an active sensor with well calibrated thermal microclimate devices. Accordingly, it was found that proactive adjustment can be conducted in order to minimize waste.

**Table:** Specifications Table

Subject area	Energy Management
More specific subject area	Indoor Environment Monitoring
Type of data	Graph of indoor environment parameters (temperature and humidity) retrieved using two types of measuring instruments: Arduino-based and Thermal Microclimate HD 32.1.
How data was acquired	Data were acquired employing two approaches; First, using the integration of the active sensor on the developmental board and second, by utilizing a well-calibrated measuring instrument namely thermal microclimate [1]: Developmental board (Arduino Uno) with sensor integration: Temperature Humidity sensor module DHT11 [2] Measuring instrument: Thermal Microclimate HD 32.1
Data format	Raw
Experimental factors	The room that was selected to collect the data collection had fixed operating hours, between 9.00 a.m. and 5.00 p.m. daily. The number of occupants working in the room fluctuated and was random given the rapid and active movement of occupants moving in and out of the room. The dimension of the room was approximately 61.91 m^2^ having a minimum occupancy of 10 people and with a maximum occupancy of 22 people. The AHU controlled the room conditioning unit. Although, the operation of the AHU was presently affected by the insufficient supply by the chiller units since two out of three chiller units had experienced breakdowns.
Experimental features	The devices for monitoring were set up with the sensor to measure and record both temperature and humidity. All related sketches were uploaded to the developmental board before configuring the device.
Data source location	Human Resource Department, Chancellery Building, Universiti Teknikal Malaysia (UTeM), Durian Tunggal, Melaka, Malaysia. Coordinate: (102.19E, 2.18N).
Data accessibility	Data within this article
Related research article	A. Ngah Nasaruddin, B.T. Tee and M.T. Musthafah, (2019). An Integrated Approach of monitoring Indoor Environment Inside Building. Proceedings of Mechanical Engineering Research Day 2019, (MERD′19). pp. 95–97.

**Table:** 

**Value of the Data** • These data would provide valuable insight into multiple monitoring platform alternatives tested in a building indoor environment condition of academics office conditions. • These data enable other researchers to offer better justification regarding monitoring the environment platform between an open source and well-calibrated instrument based on data accuracy, feasibility and reliability. • These data provide the opportunity for facilities managers to review and identify defective working conditions and operation of building services for instance, the HVAC system by referring to the guidelines of the Malaysian Standard MS 1525:2014.

## 1 Data

The parameters retrieved from indoor environment monitoring include the temperature and relative humidity. The raw data were divided into two parts which is the one retrieved from Arduino based platform (Part 1) and one from the thermal microclimate (Part 2) respectively. Sample data representing 20 minutes were collected from two consecutive days and was divided into two series which is series one (S1) is from 10.07 a.m. to 10.27 a.m. and series two (S2) is from 11.07 a.m. to 11.27 a.m.. After that, both sets of data underwent quantitative analysis to attain a perspective on data reliability with the data retrieved from the thermal microclimate used as a baseline.

## 2 Experimental design, materials, and methods

**Part 1:** Arduino-based monitoring platform

**Fig. 1:**
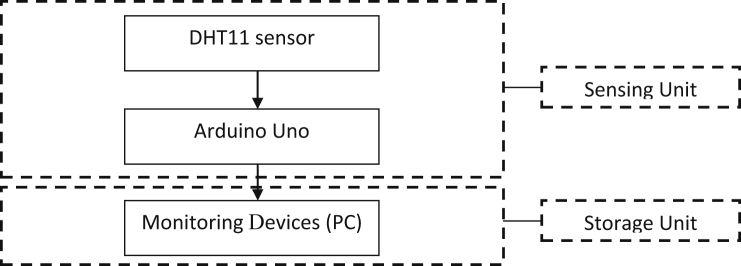
Framework of the Arduino based monitoring platform.

Fig. 1 show the framework for the Arduino based monitoring platform of the indoor environment. Notably, interoperability among the heterogeneous devices is considered to avoid packet data loss due to failed communication [1,2].

**Fig. 2:**
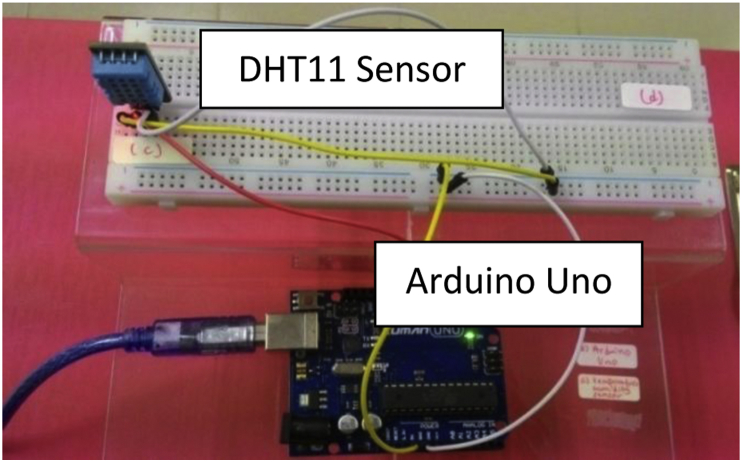
Circuit assembly.

For this case study, the sensing unit consisted of a DHT11 sensor and Arduino Uno board (ATmega328P) as illustrated in Fig. 2. The communication between the DHT11 sensor module and the Arduino board was assigned on the digital pin via serial communication [3,6]. Based on the technical specification provided by the manufacturer the DHT11 module able to provides humidity reading of the range between 20% and 90% while the temperature reading in the range between zero and 50 °C. However, accuracy is ±5% and ±2 °C for both the humidity and temperature, respectively. Notably, sensor allocation and determining the number of installed sensors in a space are important concerns for consideration regarding sensor data mapping studies [5]. Although, these two concerns are disregarded in this case study since the ultimate aim is to compare two monitoring platforms. In addition, before preliminary monitoring, it showed that no significant changes in the environment condition at different points in the room. A set of Arduino sketches was configured utilizing open-source Arduino software, Integrated Development Environment (IDE). The following algorithm describes the flow of the Arduino sketch for the sensing unit used to perform the monitoring process:

a) Include library for DHT11 sensors and communication;

b) Define input pin for sensor;

c) Reading the input from sensor and store it;

d) Define delay;

e) Return.

**Fig. 3:**
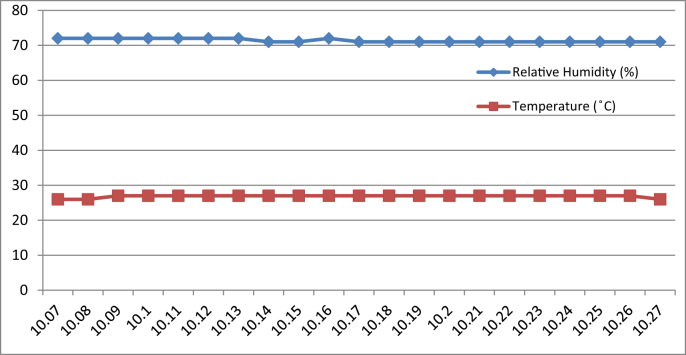
Graph of Temperature and Humidity Retrieved using The DHT11 Sensor module for S1 of Day 1.

**Fig. 4:**
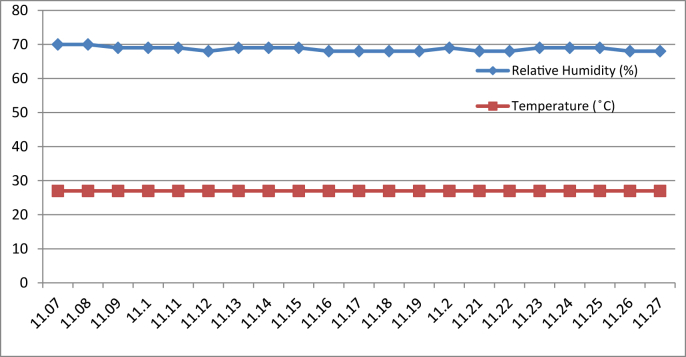
Graph of Temperature and Humidity Retrieved using The DHT11 Sensor module for S2 of Day 1.

**Fig. 5:**
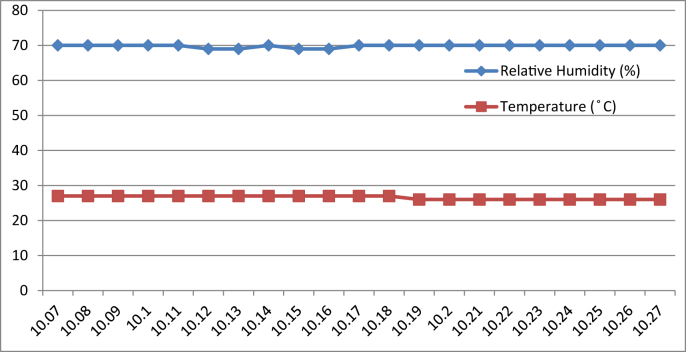
Graph of Temperature and Humidity Retrieved using The DHT11 Sensor module for S1 of Day 2.

**Fig. 6:**
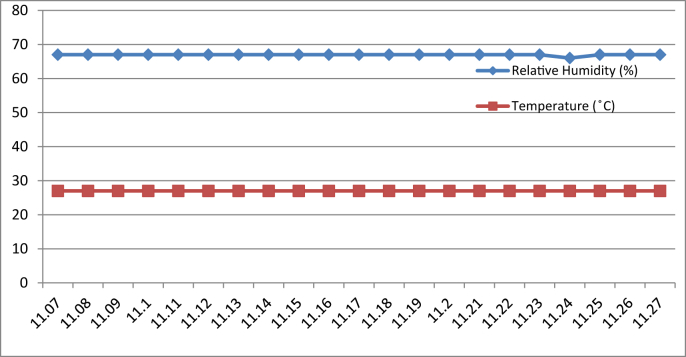
Graph of Temperature and Humidity Retrieved using The DHT11 Sensor module for S2 of Day 2.

Fig. 3, Fig. 4, Fig. 5, Fig. 6 represent the data retrieved from the Arduino based monitoring platform. Fig. 3, Fig. 4 represent the data of the temperature and humidity from day one, Fig. 5, Fig. 6 from day two, each for their respective series.

**Part 2:** Thermal microclimate measuring instrument

**Fig. 7:**
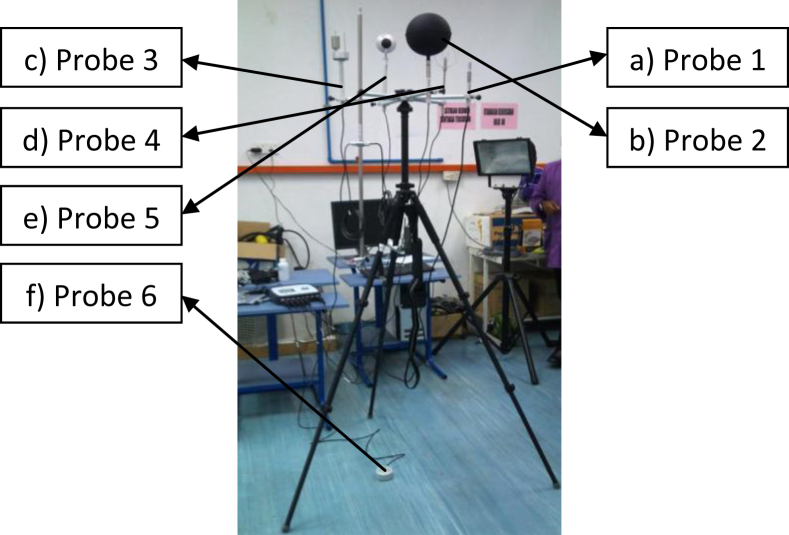
Set-up and configuration of the thermal microclimate measuring instrument.

Thermal Microclimate HD32.1 is a measuring instrument designed to analyse the microclimate in moderate, cold and hot environments. For the purpose of this case study, Program A of HD32.1 Microclimate Analysis was run in which measured the relative humidity and environment temperature (probe 1) in addition to other parameters such as the globe thermometer temperature (probe 2), natural ventilation wet bulb temperature (probe 3), atmospheric pressure (in-built), wind speed (probe 4), radiant temperature (probe 5) and floor level temperature (probe 6) simultaneously. Fig. 7 illustrates the configuration process and setting up of the thermal microclimate measuring instrument with a personal computer used to synchronize the devices. Further, the occupant parameter on thermal resistance and metabolic heat production was characterized as having a value of 081 Clo and 77.34 W/m^2^, respectively. Both thermal resistance and metabolic heat production were determined based on the normality of the occupant's daily office attire (i.e. clothing) and their activities.

**Fig. 8:**
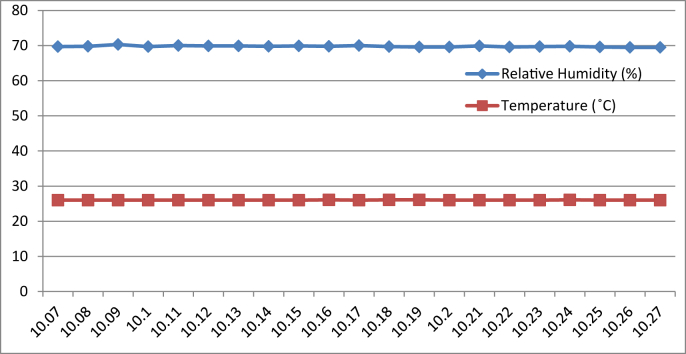
Graph of The Temperature and Humidity Retrieved using Thermal Microclimate for S1 of Day 1.

**Fig. 9:**
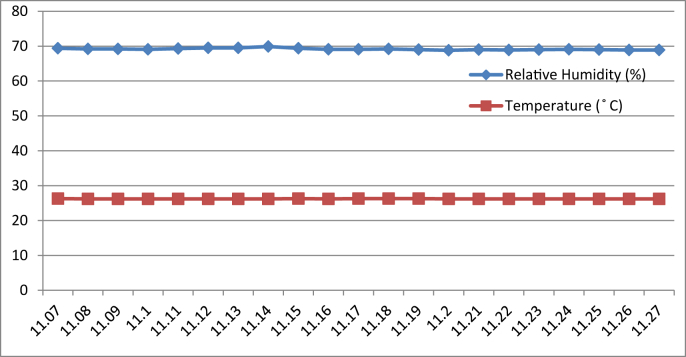
Graph of The Temperature and Humidity Retrieved using Thermal Microclimate for S2 of Day 1.

**Fig. 10:**
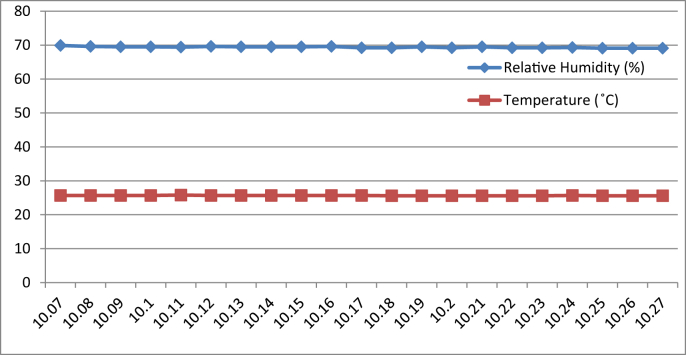
Graph of The Temperature and Humidity Retrieved using Thermal Microclimate for S1 of Day 2.

**Fig. 11:**
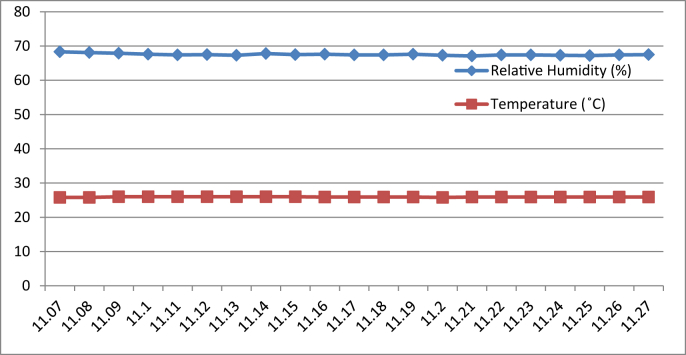
Graph of The Temperature and Humidity Retrieved using Thermal Microclimate for S2 of Day 2.

Fig. 8, Fig. 9, Fig. 10, Fig. 11 represent the data retrieved from the thermal microclimate measuring instrument. Fig. 8, Fig. 9 represent the data of the temperature and humidity from day one, and Fig. 10, Fig. 11 is from day two, each for their respective series.

Regarding of the raw data, descriptive analysis was undertaken in which involving determining information such as the mean, mode, standard deviation and skewness from the mean value. The results of the analysis are depicted in Table 1 which are arranged to compare the reading between the Arduino based platforms (C1) and the well-calibrated measuring instrument (C2) at each respective outcome of the analysis. Based on the mode pattern, the highest temperature variation recorded between the two devices was 1.6° which is the relative humidity reading in session 1 of day 1 where C1 relative humidity is leading. Further, it shows the likelihood of a fluctuation in the reading between the two devices by 1–2°. Regarding the standard deviation, the lowest for the temperature reading was 0 from C1 in session 2 of days 1 and 2 while the highest reading was 0.51 from C1 reading in session 1 of day 2. Contrastingly, the lowest value for the relative humidity reading was 0.19 from C2 in session 2 of day 1, while the highest is 0.66 from relative humidity C1 in session 2 of day 1. In addition, concerning raw data skewness, a pattern of negative skew was observed on relative humidity reading meaning that the data concentration is predominantly on the right hand side, whereas for the temperature reading, a pattern of positive skew was mostly observed. Even though a fluctuation pattern on the concentration of data was evident, it can be interpreted based on Pearson's First Skewness Coefficient in which negative skew occurs mainly since the mode is larger than the mean.

**Table 1:** Results from the descriptive analysis of raw data.

Day	Session	Reading	Outcome	Parameter	
				Relative Humidity (%)	Temperature (°C)
1	S1	C1	Mean	71.38	26.90
		C2		69.78	26.02
	S2	C1		68.67	27.00
		C2		69.17	26.22
2	S1	C1		69.81	26.57
		C2		69.39	25.66
	S2	C1		66.95	27.00
		C2		67.52	25.92
1	S1	C1	Mode	71.00	27.00
		C2		69.90	26.00
	S2	C1		69.00	27.00
		C2		69.10	26.20
2	S1	C1		70.00	27.00
		C2		69.5	25.70
	S2	C1		67.00	27.00
		C2		67.40	25.90
1	S1	C1	Standard deviation	0.50	0.30
		C2		0.19	0.04
	S2	C1		0.66	0
		C2		0.26	0.04
2	S1	C1		0.40	0.51
		C2		0.21	0.06
	S2	C1		0.22	0
		C2		0.29	0.07
1	S1	C1	Skewness	0.77	−0.32
		C2		−0.64	0.47
	S2	C1		−0.51	0
		C2		0.26	0.55
2	S1	C1		−0.47	−0.85
		C2		−0.51	−0.65
	S2	C1		−0.22	0
		C2		0.42	0.28

The ultimate goal of the data extraction was to test data stability between the DHT11 sensor module and thermal microclimate devices. The Arduino based platform and well calibrated measuring instrument were compared in a moderate statistical manner. It is well acknowledged that the accuracy of all measuring devices is exposed to degradation over time through normal wear and tear despite other factors and both types of measuring instruments succumb to experimental error and uncertainty [4].

By referring to the Malaysian Standard MS 1525:2014, the recommended design of relative humidity and dry bulb temperature is between 50% and 70% and between 24 °C and 26 °C, respectively [[8], [9]]. The results indicated that the average of both relative humidity and temperature exceeded the recommended value as recommended in the Malaysian Standard, which presented the highest recorded value of relative humidity and temperature of 71.38% (C1, S1, Day 1) and 27 °C (C1, S2, Day 1)(C1, S2, Day 2) respectively. In addition, based on the data obtained from the Thermal Microclimate HD32.1 measuring instrument, it can be concluded that the overall thermal environment was warm according to the overall dictated Predicted Mean Vote (PMV) and Predicted Percentage of Dissatisfied (PPD) value. Here, the ASHRAE Standard 55-2017 recommends PPD Lower than 10 a PMV range of more than −0.5 however less than +0.5 (−0.5 < PMV < +0.5) [7]. Since the thermal sensation was defined as warm in the measured spaces, this means that their PMV and PPD were outside the recommended ASHRAE standard range thus experiencing thermal discomfort.
